# Supplementary material for: Rapid and Simultaneous Authentication of Six Laver Species Using Capillary Electrophoresis-Based Multiplex PCR
Source: Foods. 2024 Jan 23;13(3):363. doi: 10.3390/foods13030363 (PMC10855616; doi:10.3390/foods13030363)
Supplement: Supplementary file 1 [file foods-13-00363-s001.zip › foods-2816154-supplementary.pdf]

Table S1. DNA sequencing for positive PCR products

Species	BLAST search	
	Description	Accession no. (identity)
N. dentata	*Pyropia dentata* cv. haenam chloroplast DNA, complete genome	LC521919.1 (100%)
N. haitanensis	*Neoporphyra haitanensis* voucher Pyr_001_KOR ribulose-1,5-bisphosphate carboxylase/oxygenase large subunit and ribulose-1,5-bisphosphate carboxylase/oxygenase small subunit genes, partial cds; chloroplast	KY865244.1 (100%)
N. seriata	*Pyropia seriata* Hijikuro chloroplast *rbcL*, *rbcS* genes for ribulose 1,5-bisphosphate carboxylase/oxygenase large subunit, ribulose 1,5-bisphosphate carboxylase/oxygenase small subunit, complete cds	LC505534.1 (100 %)
P. suborbiculata	*Pyropia suborbiculata* voucher PQ6 ribulose-1,5-bisphosphate carboxylase/oxygenase large subunit (*rbcL*) gene, partial cds; chloroplast	MN636850.1 (99%)
N. yezoensis	*Neopyropia yezoensis* voucher E18141 ribulose-1,5-bisphosphate carboxylase/oxygenase large subunit (*rbcL*) gene, partial cds; chloroplast	MN561423.1 (100%)
N. tenera	*Neopyropia tenera* 1477 chloroplast *rbcL* gene for ribulose-1,5-bisphosphate carboxylase/oxygenase large subunit, partial cds	LC715152.1 (100%)

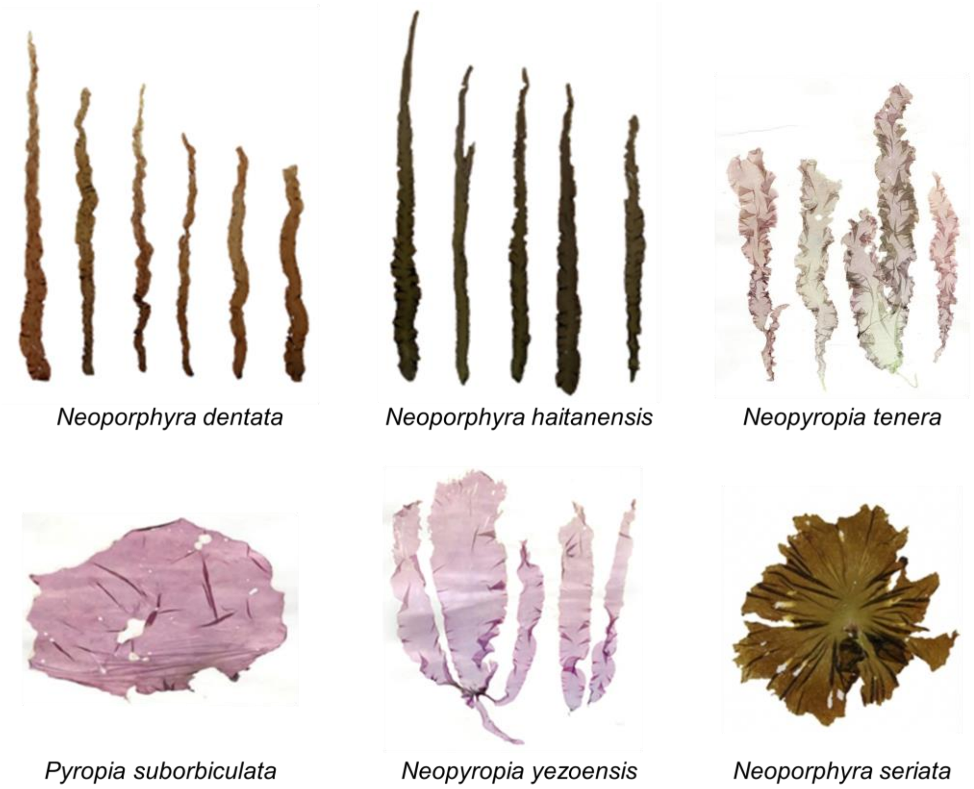

Figure S1. Phenotypic characteristics of six laver species (*N. dentata*, *N. haitanensis*, *N. tenera*, *P. suborbiculata*, *N. yezoensis*, and *N. seriata*). Phenotypic picture was obtained from the National Institute of Biological Resources (https://species.nibr.go.kr/index.do).

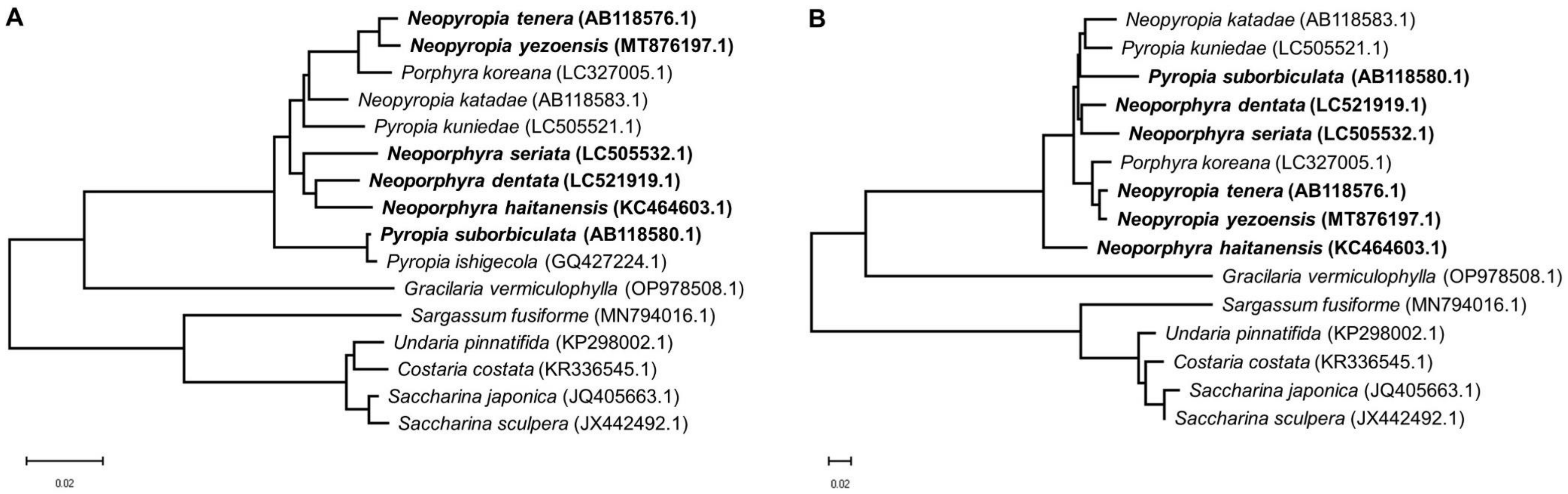

Figure S2. The dendrogram based on (A) *rbcL* and (B) *rbcS* sequences for 16 seaweed species. The phylogenetic tree was constructed by using the neighbor-joining method within the MEGA software (version 11). The bold text indicates six laver species.
